# Supplementary material for: Selectively predicting the onset of ADHD, oppositional defiant disorder, and conduct disorder in early adolescence with high accuracy
Source: Front Psychiatry. 2023 Dec 8;14:1280326. doi: 10.3389/fpsyt.2023.1280326 (PMC10739523; doi:10.3389/fpsyt.2023.1280326)
Supplement: Supplementary file 1 [file Data_Sheet_1.zip › Figure 2.DOCX]

**Supplementary Figure 2**

Receiver Operating Characteristic (ROC) curves are shown for multimodal and neural-only models of all prevailing cases of ADHD, Oppositional Defiant Disorder (ODD) and Conduct Disorder (CD) at 11-12 yrs predicted with features measured at 9-10 yrs.

|  | **Multimodal** | **Neural-only** |
| --- | --- | --- |
| **ADHD** | 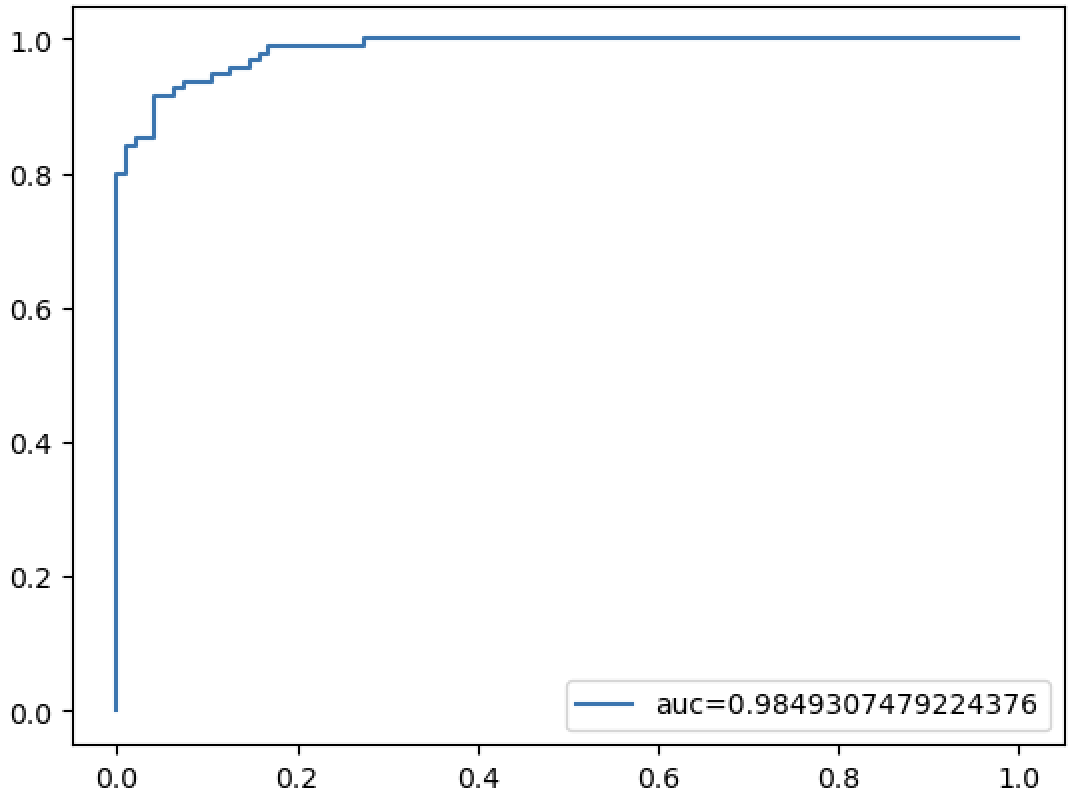 | 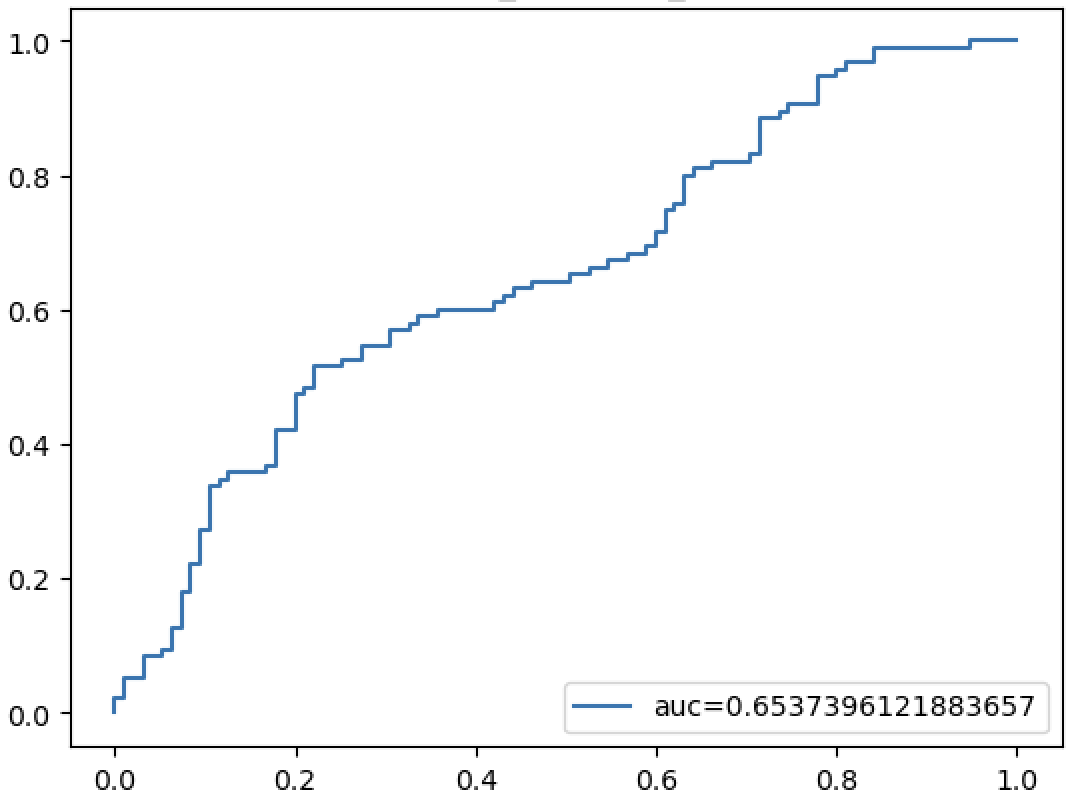 |
| **ODD** | 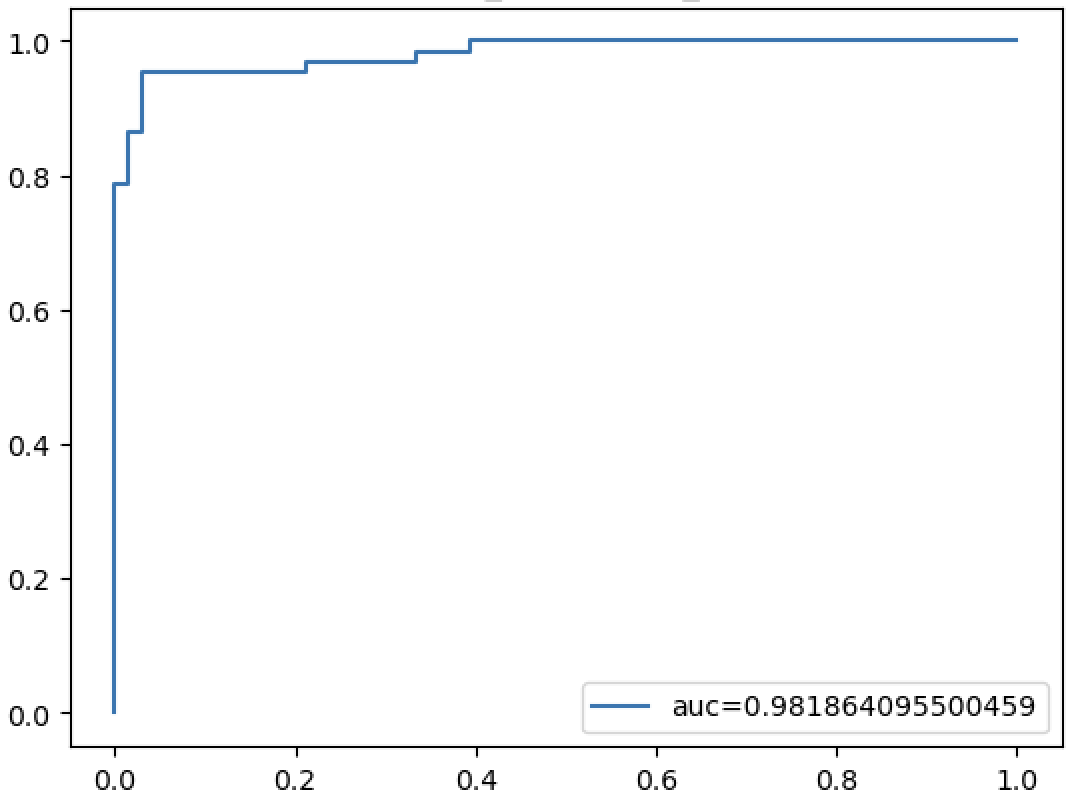 | 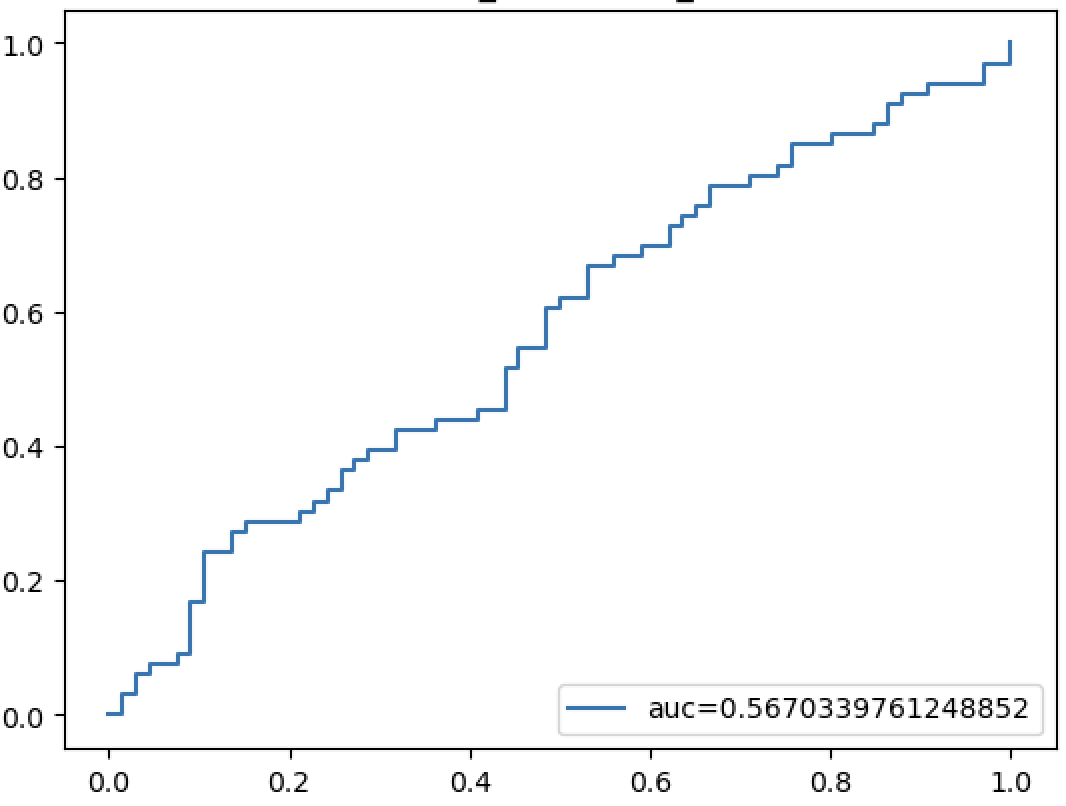 |
| **CD** | 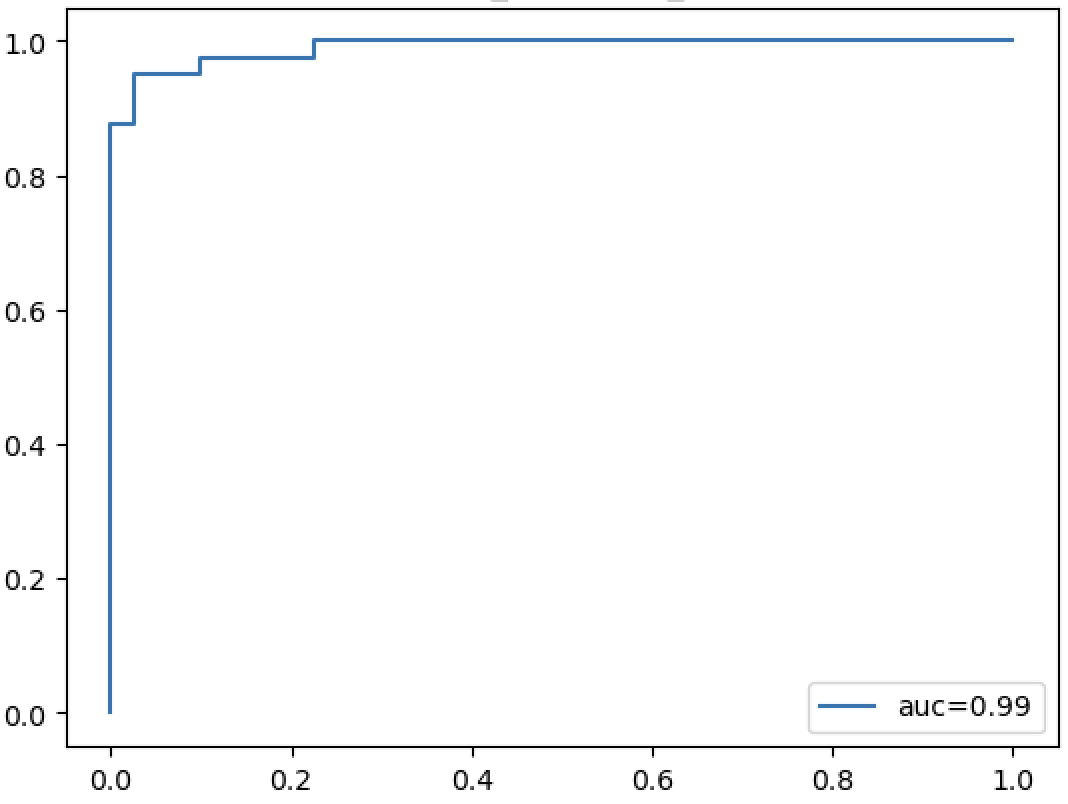 | 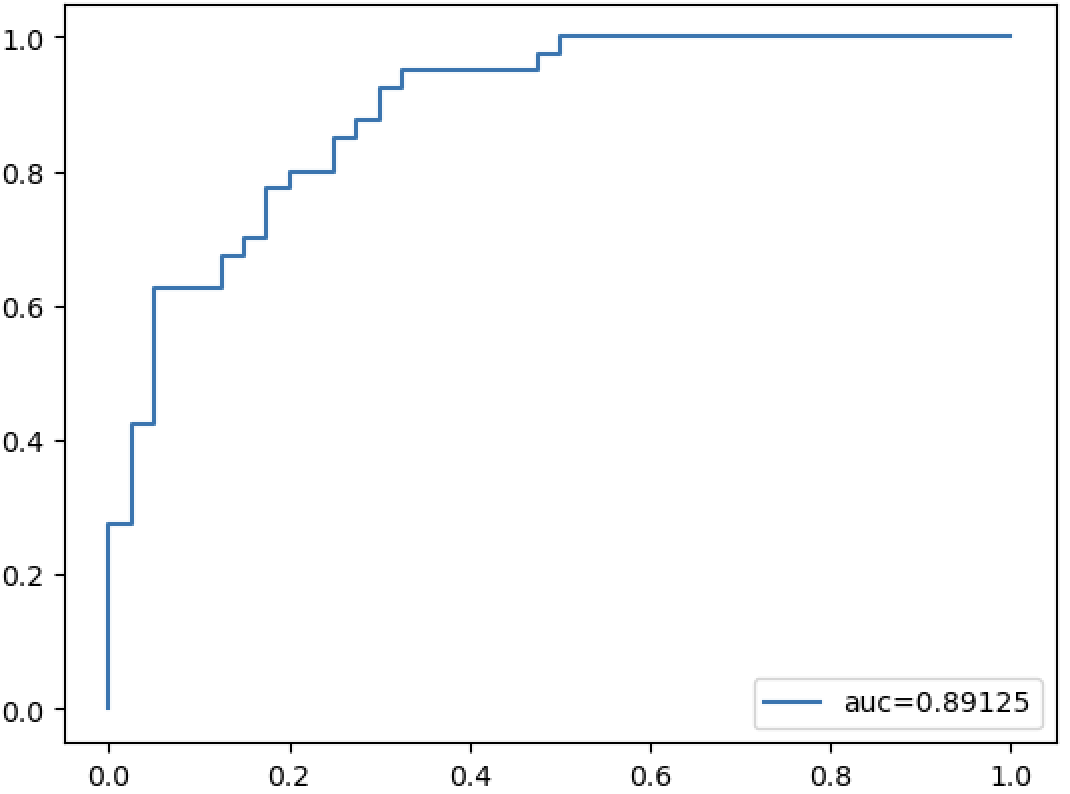 |
